# BrainGAN: Brain MRI Image Generation and Classification Framework Using GAN Architectures and CNN Models

**DOI:** 10.3390/s22114297

**Published:** 2022-06-06

**Authors:** Halima Hamid N. Alrashedy, Atheer Fahad Almansour, Dina M. Ibrahim, Mohammad Ali A. Hammoudeh

**Affiliations:** 1 Department of Information Technology, College of Computer Qassim University, Buraydah 51452, Saudi Arabia; 421200439@qu.edu.sa (H.H.N.A.); 421200443@qu.edu.sa (A.F.A.); d.hussein@qu.edu.sa (D.M.I.); 2 Computers and Control Engineering Department, Faculty of Engineering, Tanta University, Tanta 31733, Egypt

**Keywords:** brain MRI images, vanilla GANs, DCGANs, image generation, image classification, deep learning

## Abstract

Deep learning models have been used in several domains, however, adjusting is still required to be applied in sensitive areas such as medical imaging. As the use of technology in the medical domain is needed because of the time limit, the level of accuracy assures trustworthiness. Because of privacy concerns, machine learning applications in the medical field are unable to use medical data. For example, the lack of brain MRI images makes it difficult to classify brain tumors using image-based classification. The solution to this challenge was achieved through the application of Generative Adversarial Network (GAN)-based augmentation techniques. Deep Convolutional GAN (DCGAN) and Vanilla GAN are two examples of GAN architectures used for image generation. In this paper, a framework, denoted as BrainGAN, for generating and classifying brain MRI images using GAN architectures and deep learning models was proposed. Consequently, this study proposed an automatic way to check that generated images are satisfactory. It uses three models: CNN, MobileNetV2, and ResNet152V2. Training the deep transfer models with images made by Vanilla GAN and DCGAN, and then evaluating their performance on a test set composed of real brain MRI images. From the results of the experiment, it was found that the ResNet152V2 model outperformed the other two models. The ResNet152V2 achieved 99.09% accuracy, 99.12% precision, 99.08% recall, 99.51% area under the curve (AUC), and 0.196 loss based on the brain MRI images generated by DCGAN architecture.

## 1. Introduction

Generative Adversarial Networks (GANs) are categorized as generative models that use probability distributions to generate synthetic data [1]. GANs have two main parts, generator and discriminator; the generator works as a producer for synthetic data along with tacking random data as inputs, while the discriminator works as a classifier for the real data of the generated ones. GANs have been used widely in healthcare technology, because of their robustness and high performance. Moreover, the limited dataset has encouraged using GANs to generate the needed number of images to support training processes, such that it is highly important for getting more accurate results [2]. Detecting and classifying tumors are a significant issue in the medical domain. Therefore, many researches focus on various types of tumors especially the most critical and dangerous types.

Brain tumor is one of the diseases that are responsible for killing many people, adults, and children too [3]. Around 11,700 people are diagnosed with a brain tumor, therefore, it is mandatory to early detect tumors in the brain to increase the survival rate and improve the life expectancy by applying proper treatment and accurate diagnostics [4]. Doctors have been using several methods to diagnose the brain tumors, such as Magnetic Resonance Imaging (MRI) [5] and Nuclear Magnetic Resonance (NMR) imaging [6]. Yet, it takes time for the radiologists to segment and annotates the images manually, therefore, it is ought to use technology to help in this case, especially, in case of a lack of professionals in the domain. Machine learning has shown great achievements in several fields including image processing [2]. Deep learning, specifically, was applied in the medical industry which has proven successful by delivering more accurate results. This study focuses on classifying the MRI scan images into images that have a tumor, and those that do not. However, in domains like medical image classification, one has to have enough dataset points—in this case, MRI scan images—which reduce the error rate and lower the certainty of injury or death. Accordingly, data augmentation is required to increase the instances of trainable images, and thus, increase the classification accuracy.

The main contributions of this study are:

1. An expanded Brain MRI dataset that involves around 1400 images using two GAN architectures: Vanilla GAN (original GAN) and Deep Conditional GAN (DCGAN). The expanded dataset will enable us to develop more general and accurate deep learning models for diagnosing brain MRI images for tumors.
2. A framework, denoted as BrainGAN, for generating brain MRI images using multiple GAN architectures. This framework can be considered a guide for future experiments in terms of GAN architecture and parameters’ configurations. To the best of our knowledge. Generating two MRI dataset samples allows comparisons between the different GAN architectures in generating brain MRI images that are more similar to the real images.
3. A novel approach to automatically validate the images generated by GANs. Although manual validation may be more accurate, however, it is time-consuming and may not be practical due to the limited availability of MRI radiologists. Thus, this study proposes an automatic validation of generated images using deep transfer learning models, i.e., three models. The validation is performed by training the deep transfer models with the generated images by the two GAN architectures, i.e., Vanilla GAN and DCGAN, and then evaluating their performance on a test set composed of real brain MRI images.

The remaining of the paper is outlined as follows: Section 2 presents a summary of the literature review, Section 3 describes BrianGAN, the proposed framework, and Section 4 explains the experimental setup conducted to create the BrainGAN framework including the dataset of the study, image augmentation using Vanilla GAN and DCGAN, and the deep learning proposed classification models. Section 5 presents the results and discussions while Section 6 illustrates the comparative analysis and discussion. Finally, Section 7 concludes the paper.

## 2. Literature Review

In this section, we introduce some related studies with GANs and brain tumor. Many studies have been founded on the basis of GANs in the medical imaging domain. Some of these will be mentioned below.

The study by Changhee Han and others [7] under the name Combining Noise-to-Image and Image-to-Image GANs: Brain MR Image Augmentation for Tumor Detection shows that applying two-step GANs to detect a tumor in the BRATS dataset has boosted the sensitivity of the model from 93.67% to 97.48%. The same dataset was used in another study in the same year (2019) by the same main author along with other authors [8] in another study has used the Conditional Progressive Growing of GANs (CPGGANs) model which improved the accuracy by improving by 0.64%, yet the test accuracy decreases with almost 100% of sensitivity and 6.84% of specificity, this is because the classifier recognizes the synthetic images.

On the other hand, a study in the year 2019 by Han and others [9] shows that the Conditional Progressive Growing of GANs (CPGGANs) model has boosted the sensitivity by 10% in the diagnosis with clinically acceptable additional False Positives. In 2020 the Enlarged Training Dataset by Pairwise GANs for Molecular-Based Brain Tumor Classification study by Ge and others [10] applies the pairwise GANs which is a sufficient choice when the dataset is small to be used by deep learning models. The study used classification on the dataset images, in addition to, the combination of both the dataset images and augmented images, the latter has achieved higher performance.

The highest accuracy achieved by far is reached 98.57% by a model suggested by Ghassemi and others [11] which is using a pre-trained GAN model that was applied to two datasets, Nanfang Hospital and General Hospital MRI brain images and Tianjin Medical University in China for the years from 2005 to 2010. From the results, it can be seen that this proposition has significantly improved the overall efficiency.

In the previous year, a study by Chenjie and others [12] aims to detect tumors of brain MRI after applying a multi-scale gradient GAN (MSG-GAN) algorithm. The dataset under study consisted of 231, 499, and 306 images from meningioma, glioma, and pituitary tumors classes. After applying the concept of image synthesis and classification on images using deep convolutional neural networks (CNNs) the model accuracy has reached 88.7%. This has demonstrated that the MSG-GAN model has achieved its functionality of producing images that are close to the real images of the dataset. The same goal for another study in the same year by Sivadas, Deepak, and Ameer [12] using the Progressive Growing of GANs (PGGANs) algorithm achieved 91.08%. This could be owing to the fact that the study used Res-Net 50 to classify the synthesized images. This study has used the BRATS dataset, which has 220 patient images divided into 154 images used in the training of the model, 44 images for the validation, and the rest (22 images) used in the testing process.

A recent study which was conducted in 2021 by Changhee and others [13] and named MADGAN: unsupervised medical anomaly detection GAN using multiple adjacent brain MRI slice reconstruction aims to detect anomalies in the MRI slices, the study was able to detect anomalies using the Area Under the Curve (AUC) with 0.727 for the early stage of Alzheimer’s disease and 0.894 for the mild cognitive impairment. Moreover, the study detected brain metastases on the image scans with AUC 0.921.

In the same year, another algorithm called faster Regional CNNs was suggested by Sandhiya and others [14] to identify tumors in the MRI brain images, this new approach has allowed the model to reach 93% classification accuracy which is higher than the previous studies on the same dataset. The study objective was to detect a tumor in an optimized way, mainly to detect meningioma, glioma, pituitary tumors from the MRI brain images. The optimization step comes from applying the faster CNN model to the dataset, this new approach has been practiced into three levels of detection ratings, 89.23% and 96.28% sensitivity for detecting glioma and pituitary tumors, respectively.

The result of this study [15] shows that the Glioma and Pituitary categories were more accurate than Meningioma, due to fewer numbers of images under the Meningioma category in the dataset.

A different technique has been used in [16], where the authors changed the MRI to the gray-scale image as a preprocessing step then segmentation used FC algorithm to classify a tumor and non-tumor images, after which feature extraction was performed to identify the tumor shape and position. Finally, classifying the abnormality features in the brain as a tumor, stroke, inflammatory disease, and degenerative. The result of this technique succeeds in segmentation, classification, and determining the severity of the tumor. But it fails in identifying the solid and necrotic tumors.

Also, Devanathan and Kamarasan proposed the RN-OKELM technique that performs two steps of pre-processing the images such as image resizing and data augmentation, the tumor identification in the brain image is conducted by morphological operations. Furthermore, the feature extractor is applied by the residual network (Res2Net) model and the classification is employed via OKELM model. Moreover, the parameter of OKELM has been tuned effectively to WSA to improve the performance [17].

In this study the authors classified the brain tumor into four categories (glioma, meningioma, pituitary, and no tumor) by applying EfficientNet using min-max normalization, they used Gaussian and Laplacian filter and fuzzy thresholding to perform a pre-processing step, then the augmentation process with a dense-CNN model to enhance training [18].

Dhaniya et al. combined simple k-implies with SVM to get lower order error and determine the tumor region by consolidating inherent picture structure progression and factual order data [19] While this study focus on increasing the dataset size by using Cyclic Generative Adversarial Networks to detect and classify tumor to the four categories [20]. Below we provide a summary of each of the studies listed in Table 1.

Moreover, authors in [24] present a semantically consistent GAN framework in which class identities of image segments in the source domain are used to define semantics as they are called Sem-GAN framework. Their proposed framework includes consistency constraints on the translation task that, along with GAN loss and cycle-constraints, enforces that translated images inherit the appearances of the target domain while (approximately) maintaining their identities from the source domain. Sem-GAN improves the quality of translated images by more than 20% on the FCN score, according to their experiments. Semantic segmentation models trained with Sem-GAN images produce better segmentation results than other variants. Their results show that semantic consistency, as proposed in this paper, is crucial for translation quality.

For a no-reference stereoscopic image quality assessment, the authors propose StereoQA-Net [25]. StereoQA-Net is an end-to-end dual-stream interactive network with left and right view sub-networks. LIVE stereoscopic image quality databases are used to evaluate our method. The proposed StereoQA-Net outperforms state-of-the-art algorithms on symmetrically and asymmetrically distorted stereoscopic image pairs. StereoQA-Net can predict local perceptual quality in general. Cross-dataset experiments show the algorithm’s generalizability.

The authors in [26] propose an Unsupervised Deraining Generative Adversarial Network (UD-GAN) to solve the problems by introducing self-supervised constraints from unpaired rainy and clean images. Rain Guidance Module (RGM) and Background Guidance Module (BGM) are designed to take advantage of rainy image characteristics. UD-GAN outperforms state-of-the-art methods on various benchmarking datasets.

## 3. BrainGAN: The Proposed Framework

In this study, we propose BrainGAN, a framework for generating brain MRI images based on Vanilla GAN (The original GAN is called a vanilla GAN) and DCGAN and automatically validating the generated datasets using deep transfer learning models. The validation is mainly based on the original images and the synthetic ones.

**Figure 1:**
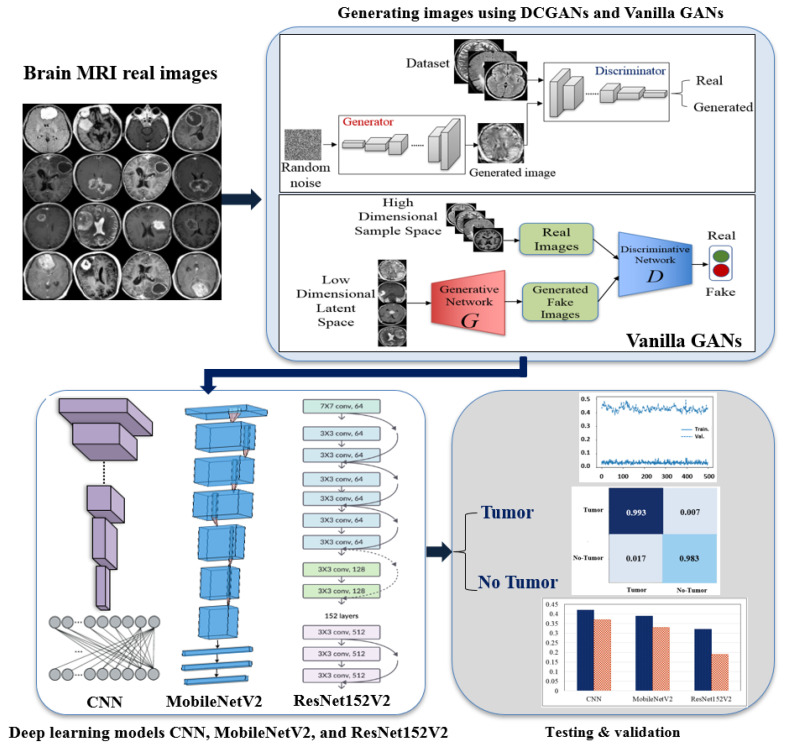
Our proposed BrainGAN framework starting with Brain MRI dataset real images, generating images using DCGAN and Vanilla GAN, deep learning models CNN, MobileNetV2, and ResNet152V2, and finally the testing & validation.

**Figure 2:**
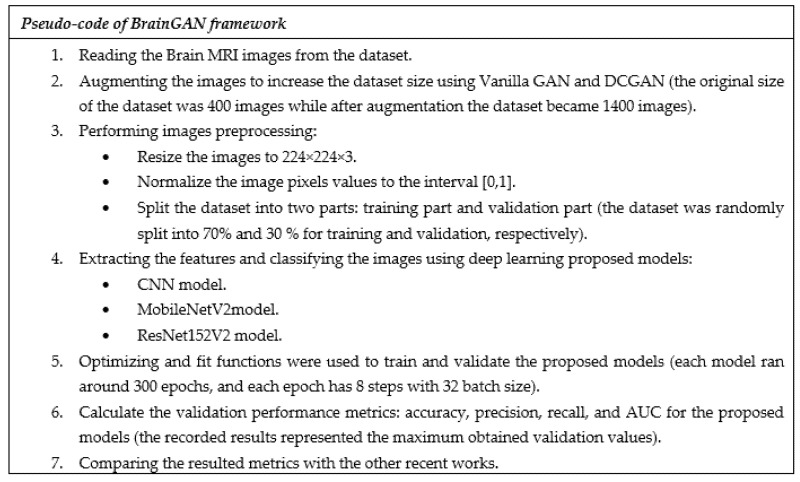
The Pseudo-code of the proposed BrainGAN framework.

BrainGAN framework is shown in Figure 1 and contains four main phases: (1) Dataset Collection, which aims to collect a dataset containing Brain MRI real images. As our image validation phase is based on image classification, it was essential to have additional classes besides the Brain tumor class., (2) Image Generation using Vanilla GAN and DCGAN to increase the number of images, (3) Multiple deep transfer learning models, i.e., CNN, MobileNetV2, and ResNet152V2, are applied to automatically validate the generated images resulted by the Vanilla GAN and the DCGAN. and (4) Generated Image Validation. Definitely, the model training is performed using the generated images of the two classes (Tumor and No tumor), and then the model testing is performed using the original images. By doing so, conclusions about the similarity between the real and generated images can be made. The proposed framework steps are summarized in Figure 2.

## 4. Experiment

### 4.1. Datasets of the Study

**Figure 3:**
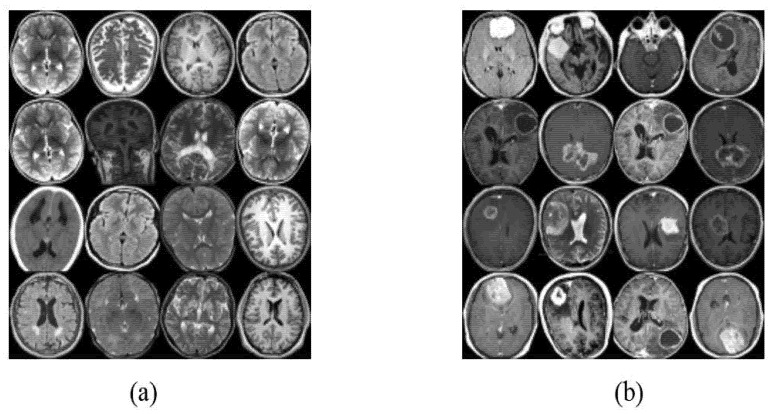
MRI scan images for two classes (**a**) No tumor samples images, and (**b**) Tumor sample images.

There are several types of brain tumors, for example, benign, malignant, and pituitary tumors among others [27]. The dataset consists of 400 MRI images divided into 170 images for a normal class, and 230 MRI images that contain cancer. This dataset was obtained from Kaggle [28], which is a data science competition platform. The dataset is organized into one folder (Braintumorimages) and contains two subfolders for each image category (Normal/Tumor) with 400 magnetic resonance (MR) images. Specifically, it contains 170 Normal MRI images and 230Tumor MRI images. Figure 3 consists of 16 MRI scan images for two classes, images that have tumors and others with no tumor, respectively.

### 4.2. Image Augmentation Using Vanilla GAN and DCGAN

Every neural network learning model uses several techniques like forwarding pass and backpropagation in finding the probability distribution that best represents the data. In fact, deep learning models were able to find this probability distribution in more complicated data, for example, audio and images. Regrettably, these concepts of neural network models did not succeed in the applications of deep generative models as they did for the previously mentioned models. This is due to the difficulty of approximating the maximum likelihood since it has multiple probabilistic computations. The Generative Adversarial Networks (GANs) study proposed architecture includes two models, the first one is called the generator G, and the second one is called the discriminator D [29] The latter is a predictive model, which is more common in machine learning where the model learns the conditional probability of the target variable given the input variable.

When the generator wants to create new variables, afterwards, it uses the Bayes theorem to calculate the conditional probability of target variable given the input variable. Hence, in the generator, the model learns the distribution of the data, and thus, generates new fake data points.

On the other hand, the discriminator classifies the data points as original or fake (which are the data points created by the generator). Therefore, these two models work in an adversarial setup, whereby they compete to get a better job. The objective of these models is that the generator attempts to maximize its probability of generating data points that are close to the original data points as much as possible, while the discriminator attempts to reduce the probability of the generator.

**Figure 4:**
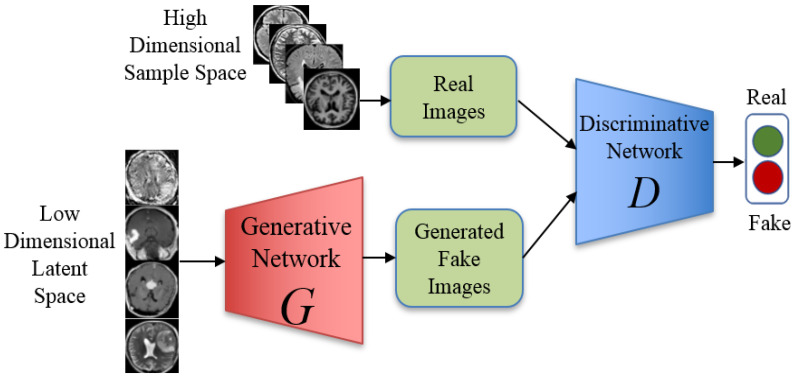
The proposed architecture of the Vanilla GANs to generate MRI images.

The architecture explained in Figure 4 is called Vanilla GANs, which is the simplest design for a generator model. It contains two models, the generator that is named the generative network. The generator gets low dimensional latent space, which is the noise to construct new images or data points. Now, both the real images and the generated fake images are passed to the discriminative network which decides if the image is real or not [30].

The generator network *G* is in charge of receiving an input *z* and producing synthetic visuals using random noise (*z*). On the other hand, the discriminator network *D* receives data from two different sources as its inputs: the real dataset’s original images, which are denoted by *x*, and the synthesized images, which are denoted by *z* [31,32]. The discriminator *D* works toward the maximization of a function known as the loss function, whereas the generator *G* works toward the minimization of that function in the following manner:

(1) $$\begin{array}{c} \min\\ G \end{array}\;\begin{array}{c} \max\\ D \end{array}\;V\left(D,G\right)=E_{x∼Ρdata\;\;}\left(x\right)\left[\log D\left(x\right)\right]+E_{z∼Ρz\;\;}\left(z\right)\left[\log\left(1-D\left(G\left(z\right)\right)\right)\right]$$

where D(x) is an estimate provided by the discriminator of the probability that real data instance x actually exists, G(z) is the output of the generator when noise z is inputted, $E_{x\;}$ is the value that should be expected based on all actual data instances, D(G(z)) estimate by a discriminator of how likely it is that a fake instance is real, and $E_{x}$ is the expected value of all of the random numbers that were put into the generator.

**Figure 5:**
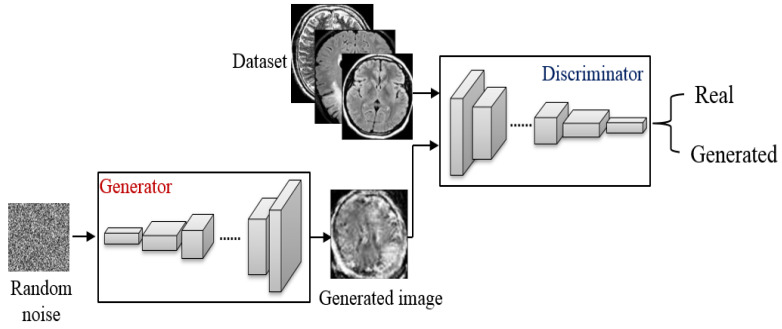
The proposed architecture of the DCGANs to generate MRI images.

The Deep Convolutional Generative Adversarial Networks or DCGANs, in short, is a different architecture that overachieved the GANs architecture in the study proposed by Alec Radford, Luke Metz and Soumith Chintala in 2016 [33]. The main idea of DCGANs is to use the convolutional-transpose layers in the generator and the convolutional layers in the discriminator. In the generator, also there are batch norm layers and ReLU activations, which with the convolutional transpose allow transferring the latent space which is derived from the normal distribution of the data, as illustrated in Figure 5.

**Figure 6:**
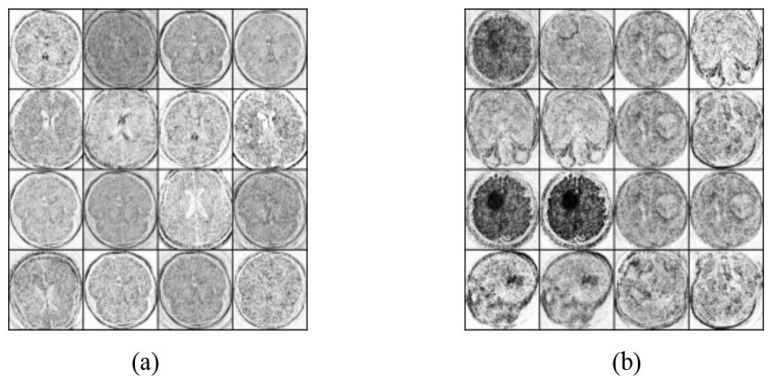
MRI scan images that are generated by applying Vanilla GANs for (**a**) Vanilla GANs no tumor images, and (**b**) Vanilla GANs tumor images.

**Figure 7:**
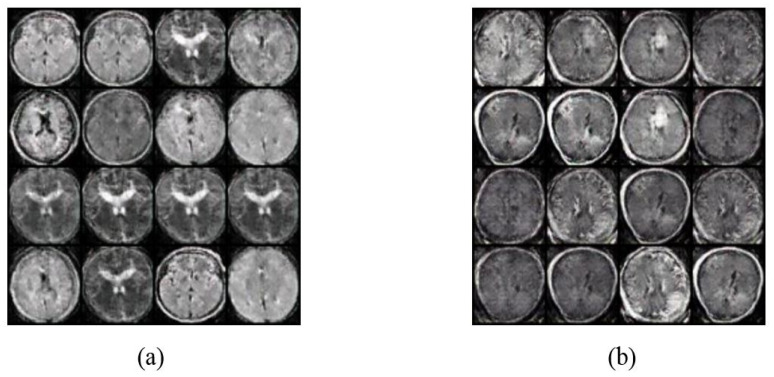
MRI scan images that are generated by applying DCGANs for (**a**) DCGANs no tumor images, and (**b**) DCGANs tumor images.

As discussed, the classification method was applied to the dataset after applying augmentation using Vanilla GANs and DCGANs. After applying the Vanilla GANs method new images were obtained, with total 1400 images classified as 700 MRI scan images that have a tumor and 700 MRI scan images with no tumor. Figure 6 demonstrates 16 MRI scan images that are generated by applying Vanilla GANs for both no tumor and tumor class. Moreover, Figure 7 demonstrates 16 MRI scan images that are generated by applying DCGANs for both tumor and no tumor classes.

Both models were run for 1000 epochs, and they took more than 12 h to run on a high-performance personal computer. We use the early stopping technique in choosing the number of epochs by set the number of epochs to a very high number and we turned off the training when the improvement over the next epochs was not satisfying and did not meet our expectations. To generate brain MRI images, the Vanilla GAN and the DCGAN were trained with the training options presented in Table 2. To get benefit of the DCGAN features, there should be multiple classes in the dataset. In our dataset, we have two classes: tumor and no tumor.

### 4.3. Deep Learning Proposed Classification Models

As machine learning focuses on solving various problems like regression, reinforcement learning, and classification, the last-mentioned technique can be applied to numerous classes of data including images [34]. The classification problem is a type of supervised learning class of machine learning, which is to say that input and output samples should be available to be provided to the machine learning model for the training process. This type of dataset is called labeled data [35]. The goal of supervised learning is to find the mapping function between the input and output. Following that, a testing process will be passed to the model to see how well it learns; this is performed by providing the model with inputs and letting it predicts their labels.

The CNNs models which are used in the image’s classification aim to understand the features of the provided images with their labels and use them to identify the labels of the images in the testing process. This study applies CNN, MobileNetV2, and ResNet152V2 to classify MRI images in the dataset augmented by Vanilla GANs and DCGANs.

The metrics utilized in this study are the most common ones, which are accuracy, precision, recall, loss, and AUC, which are often used for evaluating multi-label classifiers [36]. All these metrics depend on true positive (TP) and true negative (TN) which denote the number of negative and positive instances that are correctly classified, and false positive (FP) and false-negative (FN), which denote the number of misclassified negative and positive instances. In this study, the following measures will be used to evaluate the deep transfer learning model. The formulas of the performance metrics used in this study are presented below:

(2) Accuracy =(TP + TN)/(TP + FN + TN + FP) 

(3) Precision= TP/TP + FP 

(4) Recall= TP/TP + FN 

As a feature extraction and classification method, deep neural networks using convolutional neural networks (CNNs) are used to determine the classification of brain MRI. It is composed of three layers: input, feature extraction, and classification. The input layer contains a breast image measuring 224 × 224 × 3. Four CNN blocks comprise the feature extraction section. Each of these blocks is composed of three layers: convolution, batch normalization, and Rectified Linear Unit (ReLU). As illustrated in Figure 2, it may include a maximum pooling layer and a dropout layer. The result of the feature extraction step is then transmitted to the Flatten layer, which converts it to a one-dimensional data vector, the proper format for the classification dense layer. We employ two thick layers and dropout layers in this case. The final output is generated by a dense layer activated in a sigmoid fashion. Table 3 summarizes the proposed CNN model design. The total amount of model parameters is 27,430,058: 27,429,828 are trainable, whereas 230 are not trainable.

Additionally, the feature extraction models ResNet152V2 and MobileNetV2 are used. These models are capable of being taught using their pre-trained initial weights. This strategy increases training and coverage while maintaining a high level of accuracy. Following these models, reshaping, flattening, dense, dropout, a dense output layer with sigmoid activation function are performed. Their distinct architectures are depicted in Table 4 and Table 5. The ResNet152V2 has a total of 73,032,244 parameters: 72,931,029 trainable parameters and 101,215 non-trainable parameters. For MobileNetV2, the trainable and non-trainable parameters are 54,382,533: 54,359,423 and 23,110, respectively.

To investigate the performance of deep learning frameworks for brain tumor classification, the Python programming language is utilized in conjunction with Keras [37]. In the training and validation phases, Google Colab [38] makes use of the Graphics Processing Unit (GPU) runtime. The experiments were carried out on a computer device with Intel i-7 9700K 3.6 GHzCPU, 16 GB RAM, and NVIDIA GeForce RTX 2060 8 GB GPU. For the hidden and output layers, respectively, ReLU and Sigmoid activation functions are utilized. For both the training and validation phases, the number of epochs is 300, and the batch size is 32. Finally, Table 6 contains the learning rate (LR) and network parameters for each model.

## 5. Results and Discussions

**Figure 8:**
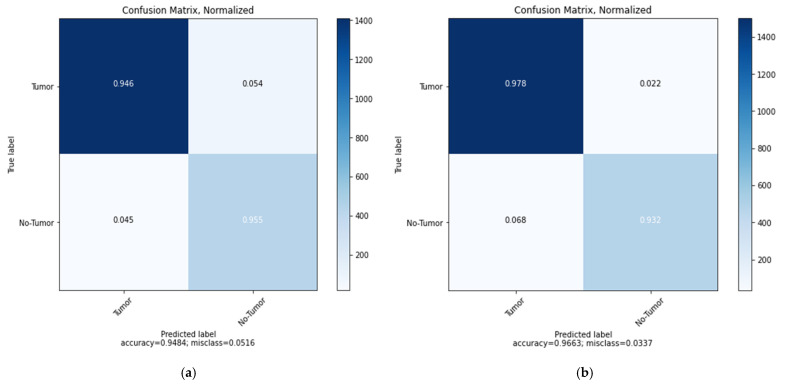
Confusion matrix for the proposed CNN model: (**a**) using Vanilla GAN image generated; (**b**) using DCGAN image generated.

**Figure 9:**
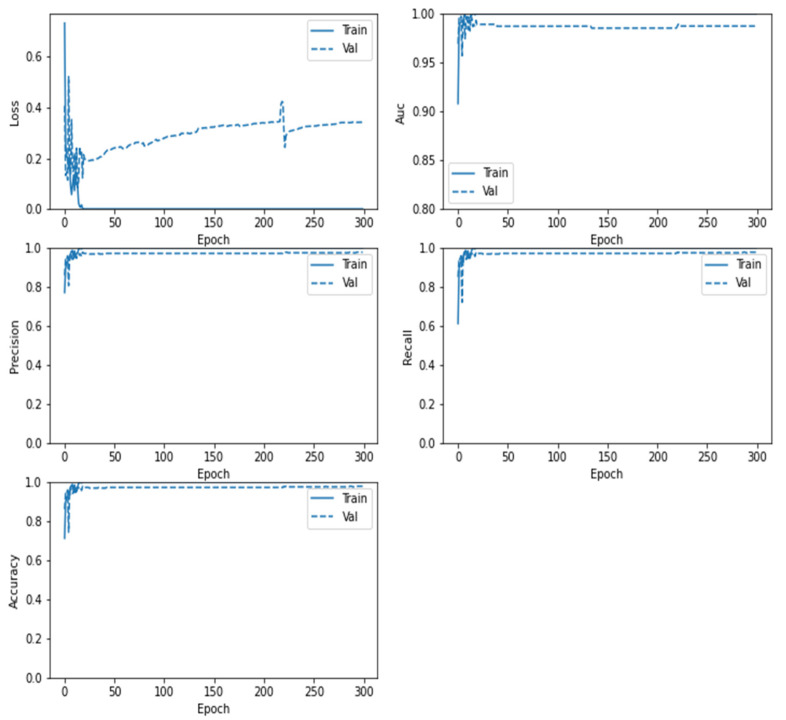
Loss, AUC, precision, recall, and accuracy between the training and validation phases with the number of epochs for the CNN model using DCGAN image generated.

This study covers the application of two main generative models, Vanilla GANs and DCGANs, with the goal to generate more MRI scan images, which increases the datasets, and therefore, gets better results. Thus, the validation of generated images is applied using deep transfer learning models CNN, MobileNetV2, and ResNet152V2 models. A confusion matrix is used to check the effectiveness of the classification and the accuracy score for the output. We build the confusion matrix for the CNN proposed model using Vanilla GAN and DCGAN generated images as in Figure 8. The figure shows that the CNN model can successfully classify the two brain status (Tumor and No-Tumor) with the highest ratio by the No-Tumor images (0.955) and total accuracy of 0.9484 and a misclass value of 0.0516 for Vanilla GAN generated images as in Figure 8a while the highest ratio using the DCGAN generated images is for the Tumor images (0.978) with total accuracy 0.9663 and misclass value of 0.0337 as in Figure 8b. This result assures that the classification is performed correctly and the DCGAN generated images outperformed the Vanilla GAN generated images. In addition, the loss, AUC, precision, recall, and accuracy between the training and validation phases are depicted in Figure 9 as a function of the number of epochs in each phase for the CNN model.

**Figure 10:**
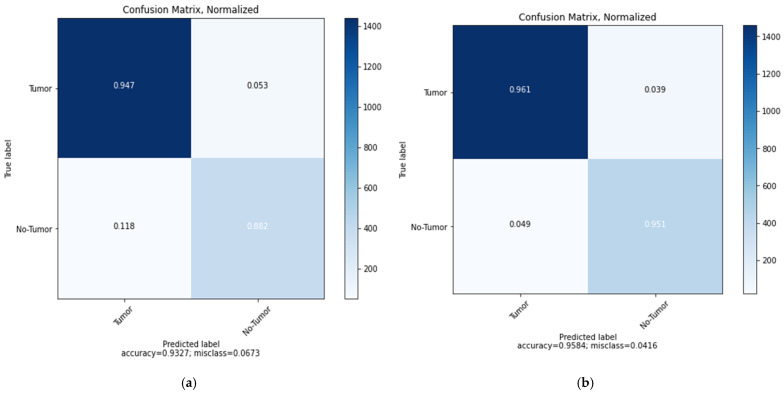
Confusion matrix for the proposed MobileNetV2 model: (**a**) using Vanilla GAN image generated; (**b**) using DCGAN image generated.

**Figure 11:**
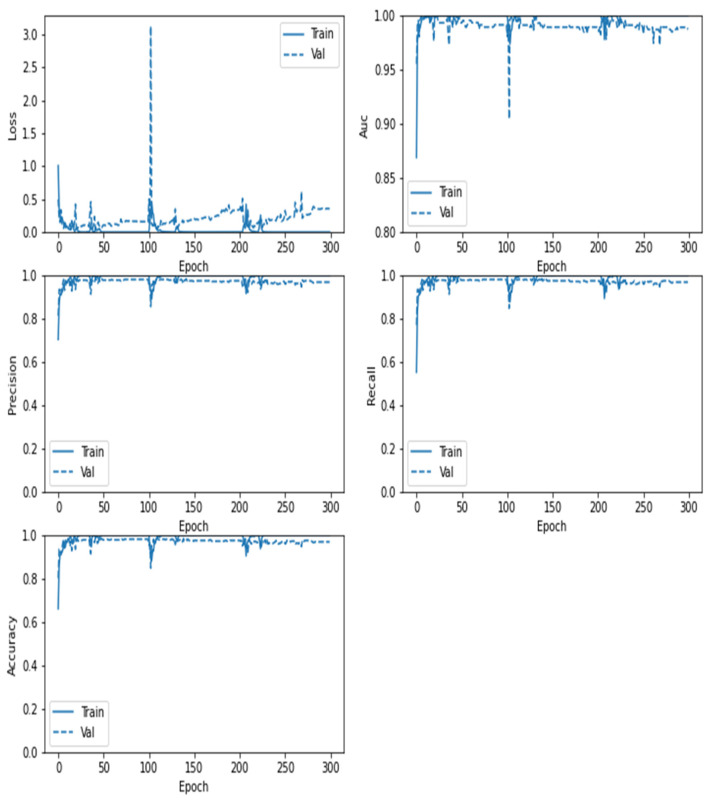
Loss, AUC, precision, recall, and accuracy between the training and validation phases with the number of epochs for the MobileNetV2 model using DCGAN image generated.

Figure 10 displays the confusion matrix for the MobileNetV2 model which demonstrates that the model can classify the two brain status (Tumor and No-Tumor) with the highest ratio to the Tumor images (0.947) and total accuracy of 0.9327 and misclass value of 0.0673 for Vanilla GAN generated images as in Figure 10a whereas the highest ratio using the DCGAN generated images is for the Tumor images (0.961) with total accuracy 0.9584 and misclass value of 0.0416 as in Figure 10b. This result promises that the classification is performed correctly and the DCGAN generated images outperformed the Vanilla GAN generated images. In addition, Figure 11 shows the loss, AUC, precision, recall, and accuracy between the training and validation phases as a function of the number of epochs in each phase for the MobileNetV2 model.

**Figure 12:**
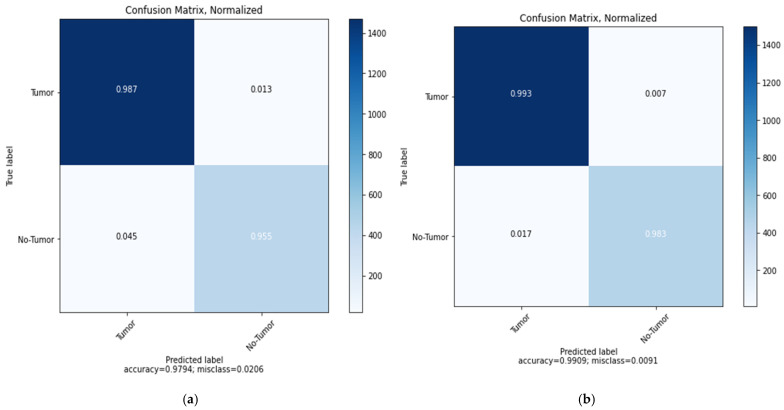
Confusion matrix for the proposed ResNet152V2 model: (**a**) using Vanilla GAN image generated; (**b**) using DCGAN image generated.

**Figure 13:**
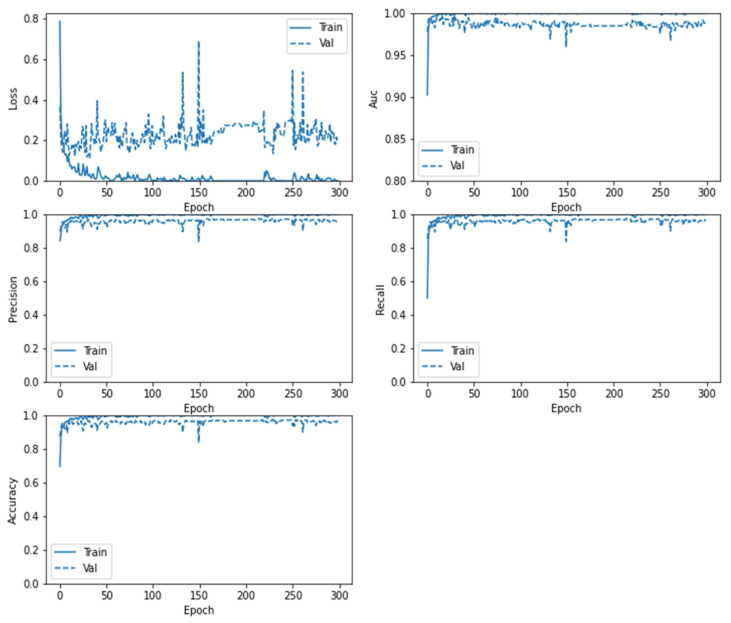
Loss, AUC, precision, recall, and accuracy between the training and validation phases with the number of epochs for the ResNet152V2 model using DCGAN image generated.

Similarly, in Figure 12, the confusion matrix of the ResNet152V2 model shows that the brain tumor and No-Tumor classification statues with the highest ratio to the Tumor images (0.987) and total accuracy of 0.9794 and misclass value of 0.0206 for Vanilla GAN generated images as in Figure 12a whereas the highest ratio using the DCGAN generated images is for the Tumor images (0.993) with total accuracy 0.9909 and misclass value of 0.0091 as in Figure 12b. For the ResNet152V2 model, Figure 13 plots the loss in AUC, precision, recall, and accuracy as a function of the number of training and validation epochs in each phase.

An interesting result that we came up, the models achieved higher accuracy when they were trained on the images generated by the DCGAN as compared to the Vanilla GAN. In particular, the ResNet152V2 had the highest accuracy, with 99.09 using DCGAN. This indicated that the generated images by the DCGAN are more similar to the real images than those generated by the Vanilla GAN.

## 6. Comparative Analysis and Discussion

The dataset contains 1400 images of the two classes: Tumor and No-Tumor brain MRI images. Each class contains 700 images. These images were generated in the previous phase using Vanilla GAN and DCGAN architectures. Three deep transfer learning models have been selected for validation. After the three models have been trained using the generated images, the models are tested with a dataset of 400 MRI real images for the two classes. Table 7 presents the performance metrics of the three validation models in terms of loss, accuracy, precision, recall, and AUC. The table presents the models’ performance once when they are trained on images generated by the Vanilla GAN and another when they are trained on images generated by the DCGAN. The bolded values show a higher value of all the metrics loss, accuracy, precision, recall, and AUC.

**Figure 14:**
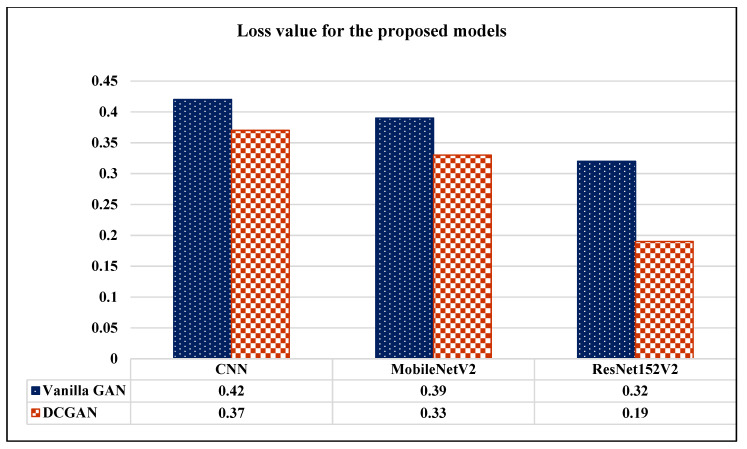
Loss measures for the CNN, MobileNetV2, ResNet152V2 models using Vanilla GAN and DCGAN image generated.

**Figure 15:**
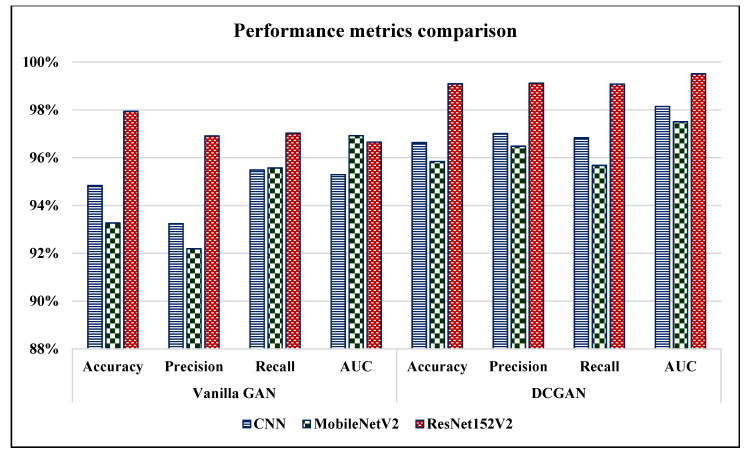
Accuracy, precision, recall, and area under the curve (AUC) measures for the proposed CNN, MobileNetV2, ResNet152V2 models using Vanilla GAN and DCGAN image generated.

Using ResNet152V2 model, we can conclude that the images generated by DCGAN for the three classes were more alike to the real images as compared to the images generated by the Vanilla GAN. As shown in Figure 14, the models achieved lower loss when they were trained on the images generated by the DCGAN as compared to the Vanilla GAN. In particular, the ResNet152V2 had the lowest loss, with 0.19 using DCGAN. Moreover, Figure 15 demonstrates that the ResNet152V2 model achieved higher values in all the metrics; accuracy, precision, recall, and AUC when they were trained on the images generated by both the Vanilla GAN and the DCGAN. In particular, the DCGAN outperformed the Vanilla GAN in the all the performance metrics.

**Figure 16:**
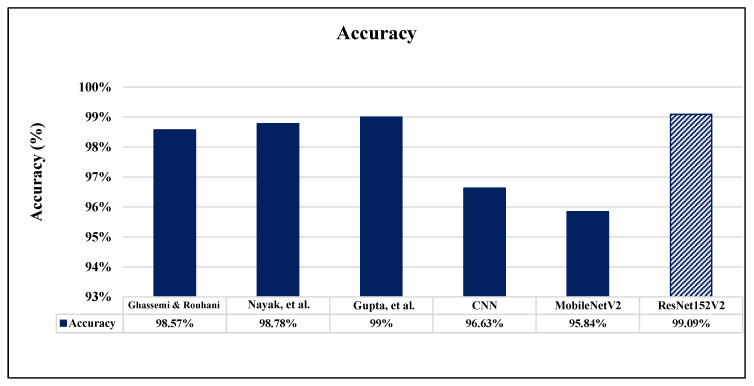
Accuracy performance metrics comparison between our proposed models and previous studies [11,18,20].

**Figure 17:**
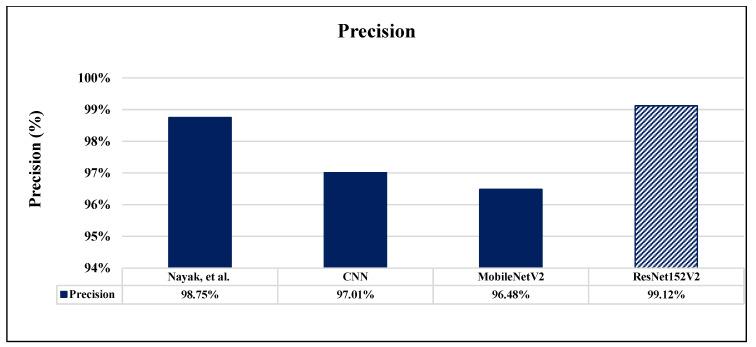
Precision performance metrics comparison between our proposed models and previous studies [18].

**Figure 18:**
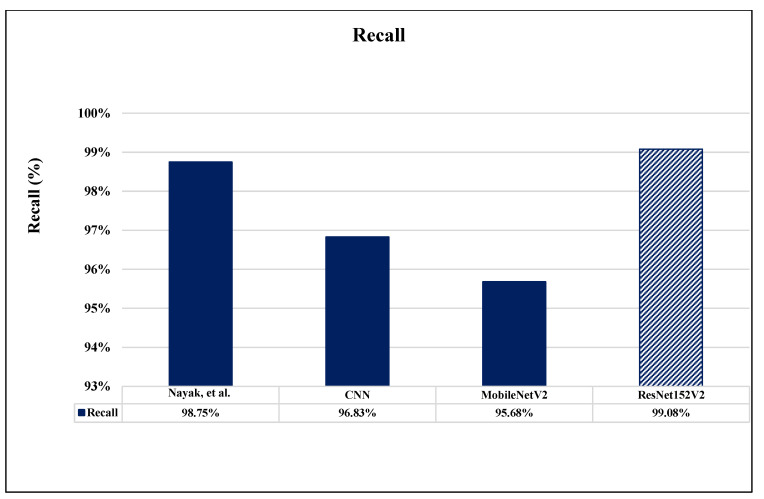
Recall performance metrics comparison between our proposed models and previous studies [18].

A comparison between the proposed completed work of this paper and the validation results of the other recent works which are introduced based on brain MRI dataset images is illustrated in Table 8. Researches [11], and [20] reported only one performance metric: accuracy. Accuracy, precision, and recall were measured in research [18]. As clearly shown in Table 8, it is evident that our proposed ResNet152V2 model achieves the highest results for all used performance measurement metrics in comparison with the previous works. Also, all our proposed models exceed the recently introduced methods in the literature. Figure 16, Figure 17 and Figure 18 illustrate the performance metrics comparison between our proposed models and previous studies for the accuracy, precision, and recall, respectively.

## 7. Conclusions and Future Work

It was proposed in this paper to use GAN architectures and deep learning models to generate and categorize brain MRI images. The framework is referred to as BrainGAN and it is described in detail. Thus, this research presented an automated method of determining whether or not the images generated are satisfactory. It employs three models: CNN, MobileNetV2, and ResNet152V2. Deep transfer models are trained with images created by Vanilla GAN and DCGAN, and their performance is then evaluated on a test set comprising of real brain MRI scans, as described in detail below. The results of the experiment revealed that the ResNet152V2 model outperformed the other two models in terms of overall performance. Based on the brain MRI pictures generated by the DCGAN architecture, the ResNet152V2 obtained 99.09 percent accuracy, 99.12 percent precision, 99.08 percent recall, 99.51 percent area under the curve (AUC), and 0.196 loss. An interesting result that we came up, the models achieved higher accuracy when they were trained on the images generated by the DCGAN as compared to the Vanilla GAN. In particular, the ResNet152V2 had the highest accuracy, with 99.09 using DCGAN. This indicated that the generated images by the DCGAN are more similar to the real images than those generated by the Vanilla GAN.

Ongoing work intends to enhance the performance of the proposed model by raising the number of images in the used datasets, increasing the training epochs and using other deep learning techniques and other GAN architectures in both classification and augmentation.

## Figures and Tables

**Table 1:** A summary of brain-related studies based on MRI images.

Ref.	Year	Classification Method	Image Type	Dataset	Performance	GAN
[7]	2019	Two-Step GANs	MRI	BRATS	Sensitivity 93.67–97.48%	√
[8]	2019	CPGGANs	MRI	BRATS	Accuracy 0.64% Specificity 6.84%	√
[9]	2019	CPGGANs	MRI	Contrast-Enhanced T1-Weighted (T1c) Brain Axial MR Images	Sensitivity 10%	√
[10]	2020	Pairwise GANs	MRI	3D Brain Volume Images from TCGA-GBM and TCGA-LGG	Average Accuracy 88.82%	√
[11]	2020	Pre-Trained GAN	MRI	Nanfang Hospital General Hospital MRI Brain Images Tianjin Medical University in China [2005–2010]	Accuracy 98.57%	√
[12]	2020	MSG-GAN	MRI	Figshare BRATS (220 Patient Images)	Accuracy 88.7%	√
[13]	2021	MADGAN	MRI	1133 Healthy T1-Weighted (T1) 135 Healthy Contrast-Enhanced T1 (T1c)	AUC 0.921	√
[14]	2021	Faster Regional CNNs	MRI	3064 T1-Weighted and Contrast-Enhanced Images Glioma 1426, Pituitary 930 and Meningioma 708 Images From 233 Patients	Accuracy 93% Sensitivity 89.23%	-
[15]	2021	VGG-19	MRI	Figshare BRATS (220 Patient Images)	Accuracy 94% F1-score 94%	-
[16]	2021	FCM-IWOA- Based RBNN Classification	MRI	Dataset 1: Kaggle [21] Dataset 2: Kaggle [22] Dataset 3: BRATS [23]	Max. Specificity of 0.945 Max. Sensitivity of 0.96 Max. Accuracy of 0.951 Max. F1-Score of 0.961 Max. Precision of 0.96	-
[17]	2022	RN-OKELM	MRI	BT (98/155 Images) Abnormal/Tumor Class.	Accuracy 97.93 Sensitivity 97.92 Specificity 97.98	-
[18]	2022	Dense EfficientNet	MRI	T1 Contrast Brain Tumors Kaggle.com. 3260 Different Types of Brain MRI Images	Accuracy 98.78% Precision 98.75% Recall 98.75%	-
[19]	2022	DA-SVM	MRI	Publicly Datasets for Tumor (Bakas et al. 2017a, b; Tobon-gomez et al. 2015).	Accuracy 89.93 Sensitivity 88.96 Specificity 88.96	-
[20]	2022	C-GAN	MRI	Publicly datasets for Tumor Detection and Classification.	Detection (Acc) 99% Classification (Acc) 98%	√

**Table 2:** Parameter configurations are used to train the Vanilla GAN and the DGAN.

Parameter	Vanilla GAN	DCGAN
Mini Batch Size	128	64
Number of Epochs	1000	2000
Discriminator Learning rate	0.0001	0.0001
Generator Learning rate	0.0002	0.0002
Optimizer	Adam	Adam

**Table 3:** The proposed CNN model architecture.

Layer (Type)	Output Shape	Parameters
conv2d_1 (Conv2D)	(None, 224, 224, 16)	438
activation_1 (Activation)	(None, 224, 224, 16)	0
batch_normalization_1 (Batch)	(None, 224, 224, 16)	64
conv2d_2 (Conv2D)	(None, 224, 224, 32)	4630
activation_2 (Activation)	(None, 224, 224, 32)	0
max_pooling2d_1 (MaxPooling2d)	(None, 74, 74, 32)	0
dropout_1 (Dropout)	(None, 74, 74, 32)	0
conv2d_3 (Conv2D)	(None, 72, 72, 64)	18,486
activation_3 (Activation)	(None, 72, 72, 64)	0
batch_normalization_2 (Batch)	(None, 72, 72, 64)	256
conv2d_4 (Conv2D)	(None, 71, 71, 128)	32,896
max_pooling2d_2 (MaxPooling2d)	(None, 24, 24, 128)	0
dropout_2 (Dropout)	(None, 24, 24, 128)	0
flatten_1 (Flatten)	(None, 73728)	0
dense_1 (Dense)	(None, 512)	27,649,248
dropout_1 (Dropout)	(None, 512)	0
dense_2 (Dense)	(None, 1000)	413,000
dropout_2 (Dropout)	(None, 1000)	0
dense_0 (Dense)	(None, 1)	1001
activation_4 (Activation)	(None, 1)	0

**Table 4:** The pre-trained ResNet152V2 model architecture.

Layer (Type)	Output Shape	Parameters
resnet152v2 (Model)	(None, 4, 4, 2048)	54,331,648
reshape_2 (Reshape)	(None, 4, 4, 2048)	0
flatten_2 (Flatten)	(None, 100352)	0
dense_3 (Dense)	(None, 256)	25,690,368
dropout_2 (Dropout)	(None, 256)	0
dense_4 (Dense)	(None, 1)	257

**Table 5:** The pre-trained MobileNetV2 model architecture.

Layer (Type)	Output Shape	Parameters
mobilenetv2_1.00_224 (Model)	(None, 7, 7, 1280)	2,257,984
reshape_2 (Reshape)	(None, 7, 7, 1280)	0
flatten_2 (Flatten)	(None, 62720)	0
dense_3 (Dense)	(None, 512)	33,113,152
dropout_2 (Dropout)	(None, 512)	0
dense_4 (Dense)	(None, 1)	513

**Table 6:** Models training parameters.

Models	Optimizer	LR	Total Number of Parameters
ResNet152V2	SGD	0.0001	54,382,533
MobileNetV2	SGD	0.0001	34,371,649
CNN	Adamax	0.00003	27,429,828

**Table 7:** Evaluation metrics for the different models using Vanilla GAN and DCGAN image generated.

Model	Vanilla GAN					DCGAN				
	Loss	Accuracy	Precision	Recall	AUC	Loss	Accuracy	Precision	Recall	AUC
CNN	0.42	94.84%	93.24%	95.49%	95.29%	0.37	96.63%	97.01%	96.83%	98.14%
MobileNetV2	0.39	93.27%	92.19%	95.57%	96.92%	0.33	95.84%	96.48%	95.68%	97.50%
ResNet152V2	0.32	97.94%	96.91%	97.03%	96.65%	**0.19**	**99.09%**	**99.12%**	**99.08%**	**99.51%**

**Table 8:** Comparison with related works.

Research	Author	Accuracy	Precision	Recall
**[11]**	Ghassemi & Rouhani (2020)	98.57%	--	--
**[18]**	Nayak, et al. (2022)	98.78%	98.75%	98.75%
**[20]**	Gupta, et al. (2022)	99%	--	--
**The proposed models using DCGAN**	CNN	96.63%	97.01%	96.83%
	MobileNetV2	95.84%	96.48%	95.68%
	ResNet152V2	99.09%	99.12%	99.08%
